# The number of circulating monocytes as biomarkers of the clinical response to methotrexate in untreated patients with rheumatoid arthritis

**DOI:** 10.1186/s12967-014-0375-y

**Published:** 2015-01-16

**Authors:** Luis Chara, Ana Sánchez-Atrio, Ana Pérez, Eduardo Cuende, Fernando Albarrán, Ana Turrión, Julio Chevarria, Angel Asunsolo del Barco, Miguel A Sánchez, Jorge Monserrat, Alfredo Prieto, Antonio de la Hera, Ignacio Sanz, David Diaz, Melchor Alvarez-Mon

**Affiliations:** Department of Medicine, University of Alcalá, Carretera Madrid-Barcelona km 33.600, 28871 Alcalá de Henares, Madrid Spain; Immune System Diseases-Rheumatology and Oncology Service, University Hospital “Príncipe de Asturias”, Alcalá de Henares, Madrid Spain; Department of Health and Medical Social Sciences, University of Alcalá, Madrid, Spain; Division of Allergy, Immunology and Rheumatology, Department of Medicine, Emory University, Atlanta, GA USA

**Keywords:** Rheumatoid arthritis, Monocyte, Methotrexate, Clinical response, Biomarker

## Abstract

**Background:**

The aim of this work was to analyze the number and distribution of circulating monocytes, and of their CD14^+high^CD16^−^, CD14^+high^CD16^+^ and CD14^+low^CD16^+^ subset cells, in treatment-naive patients with rheumatoid arthritis (RA), and to determine their value in predicting the clinical response to methotrexate (MTX) treatment.

**Methods:**

This prospective work investigated the number of circulating monocytes, and the numbers of CD14^+high^CD16^−^, CD14^+high^CD16^+^ and CD14^+low^CD16^+^ subset cells, in 52 untreated patients with RA before MTX treatment, and at 3 and 6 months into treatment, using flow cytometry.

**Results:**

The absolute number of circulating monocytes, and the numbers of CD14^+high^CD16^−^, CD14^+high^CD16^+^ and CD14^+low^CD16^+^ subset cells, were significantly higher in MTX non-responders than in responders and healthy controls before starting and throughout treatment. Responders showed normal numbers of monocytes, and of their subset cells, over the study period. The pre-treatment absolute number of circulating monocytes, and the numbers of CD14^+high^CD16^−^ and CD14^+high^CD16^+^ subset cells, were found to be predictive of the clinical response to MTX, with a sensitivity and specificity of >70% and >88%, respectively.

**Conclusions:**

Treatment-naive patients with RA showed an anomalous distribution of circulating monocyte subsets, and an anomalous number of cells in each subset. A higher pre-treatment number of circulating monocytes, and higher numbers of CD14^+high^CD16^−^ and CD14^+high^CD16^+^ subset cells, predict a reduced clinical response to MTX in untreated patients with RA.

**Electronic supplementary material:**

The online version of this article (doi:10.1186/s12967-014-0375-y) contains supplementary material, which is available to authorized users.

## Background

Rheumatoid arthritis (RA) is a highly prevalent, chronic, inflammatory disease that primarily affects multiple synovial joints [1]. Fortunately, dramatic improvements in the management of patients with RA have been achieved in the last two decades. The possibilities of controlling disease progression and joint destruction have greatly increased through the use of methotrexate (MTX) and biological drugs with anti-tumor necrosis factor (TNF) activity [1,2]. The therapeutic armamentarium for RA continues to expand, and effective new drugs are continuously being incorporated into the treatment of this disease [1]. However, there is growing evidence of wide variation in patient clinical response to current therapies [3]. The prevention of delays in the use of the most effective treatment for each patient, the avoidance of unnecessary secondary effects, and the rational use of scant economic resources have all stimulated the search for biomarkers that predict the response of individuals to different RA treatments.

Monocytes are bone marrow-derived cells that mediate essential regulatory and effector functions in innate and adaptative immunity [4]. They circulate in the blood and migrate into tissues where they differentiate into different effector cells such as macrophages, dendritic cells and osteoclasts [4-7]. Circulating human monocytes are phenotypically and functionally heterogeneous and are divided into three major subsets based on the expression of CD14 (the LPS co-receptor) and CD16 (the Fc*γ*RIII low affinity IgG receptor) [4,6-9]. Some 90% of circulating monocytes in humans are strongly positive for CD14, but do not express CD16 (CD14^+high^CD16^−^); these are known as “classic” monocytes. The remaining ~10% express CD16 plus either high or low levels of CD14 (intermediate CD14^+high^CD16^+^ monocytes and CD14^+low^CD16^+^ non-classical monocytes, respectively) [9]. There is increasing evidence that these three monocyte subsets have different functional properties, different patterns of cytokine secretion and chemokine receptor expression, different capacities to migrate into normal and inflamed tissue, and different abilities to differentiate into macrophages, dendritic cells and osteoclasts [6-8].

It is well established that the cells of the immune system play a pivotal role in the pathogenesis of the joint damage characteristic of RA, as well as in the extra-articular manifestations of the disease [1]. Monocyte/macrophage lineage cells are critical for the induction of chronic synovial inflammation and joint destruction [10]. These cells are also directly involved in the pathophysiology of the extra-articular and systemic manifestations of RA [11]. Further, patients with active RA show a marked redistribution of their circulating monocytes, with a significant expansion of those that co-express CD14 and CD16 [12]. MTX, a folate antagonist, is the most commonly used disease-modifying anti-rheumatic drug (DMARD). However, it fails to control disease activity and structural damage in some 30-40% of patients [13]. Its precise mechanism of action in the treatment of RA is unclear and, at present, no robust biomarker exits that can predict patient responsiveness to it [14].

We previously described that knowing the distribution of circulating monocytes is of value when predicting the clinical response of patients with RA to anti-TNF [15]. The hypothesis tested in this work was that the pre-treatment absolute number, distribution and migratory properties of circulating monocytes, and the numbers of CD14^+^CD16^−^ and CD14^+high^CD16^+^ subset cells, might help predict the therapeutic response to MTX treatment. The number of CD14^+high^CD16^−^, CD14^+high^CD16^+^ and CD14^+low^CD16^+^ monocytes was prospectively investigated in untreated patients with RA before initiation of MTX therapy and during the first 6 months of treatment. Monocyte changes were correlated with clinical response to treatment measured at 3 and 6 months. We found that the pre-treatment absolute numbers of circulating monocytes, and of CD14^+high^CD16^−^, CD14^+high^CD16^+^ and CD14^+low^CD16^+^ subset cells, predict the clinical response to MTX in untreated patients with RA.

## Patients and methods

### Inclusion and exclusion criteria

Fifty five patients visiting the Immunology and Rheumatology Service at the *Hospital Universitario Príncipe de Asturias* (HUPA) were enrolled in the study. All gave their informed consent to be included; the study was approved by the hospital’s clinical ethics committee. Three patients were excluded from analysis because they failed to complete the study protocol. Patients were studied in parallel with a sex- and age-matched healthy control.

#### Inclusion criteria

The entry criteria included age ≥18 years, a diagnosis of RA according to the 1987 revised European League Against Rheumatism (EULAR) criteria [16], less than 6 or 12 months since the onset of RA, a disease activity score 28 (DAS28) of >2.5 according to EULAR criteria [16], and to be DMARD-naive.

#### Exclusion criteria

The exclusion criteria were severe cardiovascular disease (congestive heart failure, uncontrolled hypertension, coronary disease, severe arrhythmia), hypercholesterolemia or diabetes mellitus, hematopoietic, lung, hepatic or renal disorders, active bacterial or viral infections, other autoimmune diseases, treatment with steroids, immunosuppressants or other drugs that interact with the immune system in the previous 6 months, possible pregnancy or lactation during the 6 month study period, simultaneous malignancy, malnutrition, and congenital immunodeficiency.

### Study protocol

All patients were treated weekly for 6 months with 10 mg MTX (orally) plus 20 mg folic acid daily for two days. The MTX dose was adjusted by increments of 5 to a maximum of 20 mg weekly until disease response criteria were met. Patients were also advised to take non-steroidal anti-inflammatory drugs at fixed doses during the study. All were monitored monthly for clinical and analytical tolerance to MTX treatment and at 3 and 6 months to assess clinical response and to undertake immunological studies. Disease activity was determined by the DAS28 score according to EULAR criteria and using a validated Spanish version of the Health Assessment Questionnaire (HAQ) [17]. The clinical response of the patients to MTX treatment was defined according to EULAR criteria for RA [16], classifying patients as responders or non-responders. The responder group included those patients with a DAS28 score of <2.6 after 6 months of MTX treatment, plus those whose DAS28 score decreased by at least 1.2 with respect to the initial value.

Three peripheral blood samples were taken from each patient by antecubital venipuncture at baseline (before starting MTX treatment), at 3 and at 6 months into treatment.

### Isolation of peripheral blood mononuclear cells

Peripheral blood mononuclear cells (PBMC) were separated out by Ficoll-Hypaque (Lymphoprep™, Axis-Shield, Oslo, Norway) gradient centrifugation [18]. They were then resuspended in RPMI 1640 (Biowhittaker Products, Verviers, Belgium) supplemented with 10% heat-inactivated fetal calf serum, 25 mM Hepes (Biowhittaker Products) and 1% penicillin-streptomycin (Biowhittaker Products). Cell enumeration was performed by conventional light microscopy using a Neubauer chamber following trypan blue dead cell exclusion criteria. The viability of fresh PBMC was checked by both trypan blue (light microscopy) and 7-aminoactinomycin D (7-AAD) (flow cytometry) exclusion.

### Immunophenotype studies

For immunofluorescent staining, fresh monocytes were incubated with a combination of fluorescein (FITC), phycoerythrin (PE), peridinin chlorophyll protein conjugate (PerCP), and Alexa Fluor-647-labeled monoclonal antibodies (MoAbs). The MoAbs were used in a four-color combination (FITC/PE/PerCP/Alexa Fluor-647): CX3CR1/CD62L/CD14/CD16. Control studies with unstained cells and cells incubated with isotype-matched irrelevant FITC-, PE-, PerCP and Alexa Fluor-647-labeled MoAbs were performed for each experiment. For these procedures, anti-CD62L, anti-CD14 and anti-CD16 were purchased from Becton Dickinson and anti-CX3CR1 purchased from MBL (Naka-ku Nagoya, Japan). Cell acquisition and four-color immunofluorescence analyses were performed using a FACSCalibur flow cytometer (Becton Dickinson) running CellQuest Pro (Becton Dickinson) and FlowJo software (Tree Star Inc, Ashland, Oregon, USA) respectively. In the FSC-SSC dot plot, a biparametric gate was drawn around the monocyte population. This gated population is displayed in a CD14-CD16 dot-plot to define the different monocyte subsets (Additional file 1: Figure S1).

### Statistical analysis

The normal distribution of the results was checked using the Kolmogorov-Smirnov test. The results of the immunophenotype study data were expressed as means and the standard error of the mean (SEM). Comparisons between patients and healthy controls, and between responders and non-responders at baseline and at the different times into treatment, were performed using the *t* test for independent samples. Comparisons between patients at baseline and after MTX treatment were performed using the *t* test for paired samples. To assess the value of baseline circulating monocytes and their different subsets as predictors of MTX treatment response at 6 months into treatment, receiver operating characteristic (ROC) curve analyses were performed, and the respective areas under the curves (AUC) determined. The best predictive cut-off value was defined as that which gave the highest product of sensitivity, specificity, positive predictive value (PPV) and negative predictive value (NPV). All calculations were performed using the Statistical Package for the Social Sciences (SPSS, version 15.0, Chicago, IL). Significance was set at p < 0.05.

## Results

### Demographic characteristics of the patients

Table 1 shows the baseline characteristics of the 37 responders and 15 non-responders included in the analysis. No significant differences in age or sex distribution were seen between these groups of patients with respect to the clinical or analytical variables studied. We also analyzed the evolution of CRP, DAS28 and HAQ in both group of patients at 6 months of follow up. In responder patients with RA, we observed a significant reduction of CRP to 5.50 ± 3.12, DAS28 to 2.18 ± 0.44 and HAQ to 0.45 ± 0.24. In non-responders patients, we also observed a significant reduction of CRP to 8.43 ± 4.22. However, the reduction of DAS28 to 3.49 ± 0.20 and HAQ to 0.72 ± 0.43 observed in non-responders were not statistically significant.

**Table 1:** **Demographic, clinical and biological data of the patients and healthy controls at baseline**

**Variables**	**Healthy controls (** ***n*** **= 15) (mean ± SD)**	**Responders (** ***n*** **= 37) (mean ± SD)**	**Non-responders (** ***n*** **= 15) (mean ± SD)**	***P*** **value**
Age (years)	46.33 ± 2.94	52.44 ± 10.90	52.08 ± 10.87	.918
Sex (men/women)	33.33%/66.67%	27.78%/72.22%	38.46%/61.54%	.351
CRP (mg/dl)		12.68 ± 7.97	15.59 ± 6.86	.251
Rheumatoid factor (+/−)		91.67%/8.33%	92.31%/7.69	.716
Anti-CCP (UI/ml)/(+/−)		445.96 ± 463.46 (89.19%/10.81%)	418.83 ± 229.47 (86.66%/13.34%)	.848
DAS28		3.38 ± .54	3.55 ± .76	.399
Erosions (+/−)		33.33%/66.67%	38.46%/61.54%	.496
Onset of symptoms (months)		12.83 ± 8.95	11.86 ± 9.49	.819
HAQ		.79 ± .52	.78 ± .49	.935

CRP, C-reactive protein; Anti-CCP, anti-cyclic citrullinated peptide antibody; DAS28, Disease Activity Score 28; HAQ, Health Assessment Questionnaire.

### Non-responder RA patients show pre-treatment increases in the total number of monocytes and in the numbers of CD14^+high^CD16^−^ and CD14^+high^CD16^+^ subset cells

The absolute number of circulating monocytes, and the numbers of CD14^+high^CD16^−^, CD14^+high^CD16^+^ and CD14^+low^CD16^+^ subset cells, were studied in all 52 treatment-naive patients before starting weekly MTX treatment, and again at 3 and 6 months.

**Figure 1:**
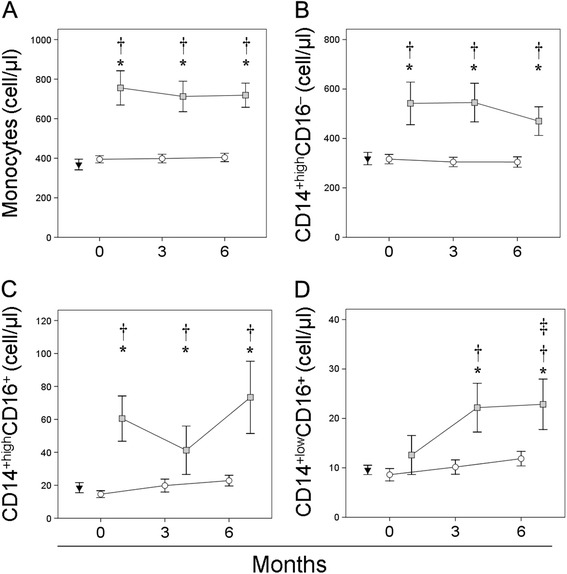
**Absolute number of circulating monocytes, and the numbers of their subset cells, in naive patients with RA at baseline and over MTX treatment.** Absolute number (cells/μl) of circulating monocytes (panel **A**), and the numbers of CD14^+high^CD16^−^ (panel **B**), CD14^+high^CD16^+^ (panel **C**) and CD14^+low^CD16^+^ monocytes (panel **D**) in non-responders (■) and responders (○) at baseline and after 3 and 6 months of MTX treatment, and of healthy controls (▼), are shown as means ± SEM. *Significant difference at baseline between groups (patients with RA and healthy controls [or responders and non-responders]). ^†^significant difference between responders and non-responders over the studied period. ^‡^Significant difference between baseline and 6 month values.

Figure 1 shows that the absolute number of circulating monocytes in the non-responders was significantly higher than in the responders at baseline. The absolute number of monocytes in the non-responders remained significantly increased with respect to responders and healthy controls over the 6 month study period. In contrast, normal numbers of circulating monocytes were found in responders at all times.

The non-responders also had significantly higher numbers of circulating CD14^+^CD16^−^ and CD14^+high^CD16^+^ monocytes than did responders and healthy controls at baseline. MTX treatment did not significantly modify the number of circulating CD14^+^CD16^−^ or CD14^+high^CD16^+^ monocytes in either group of patients. Neither were any significant differences seen between responders and healthy controls in terms of the absolute number of these cells at baseline or over MTX treatment.

No significant differences were seen between the absolute numbers of circulating CD14^+low^CD16^+^ monocytes in responders, non-responders or healthy controls at baseline. After 3 and 6 months of treatment, however, a significant increase in the number of circulating CD14^+low^CD16^+^ monocytes was observed in the non-responders, reaching values significantly higher than those recorded in responders, the values for which remained similar to those of healthy controls.

### Non-responders show progressive redistribution of the monocyte subsets

**Figure 2:**
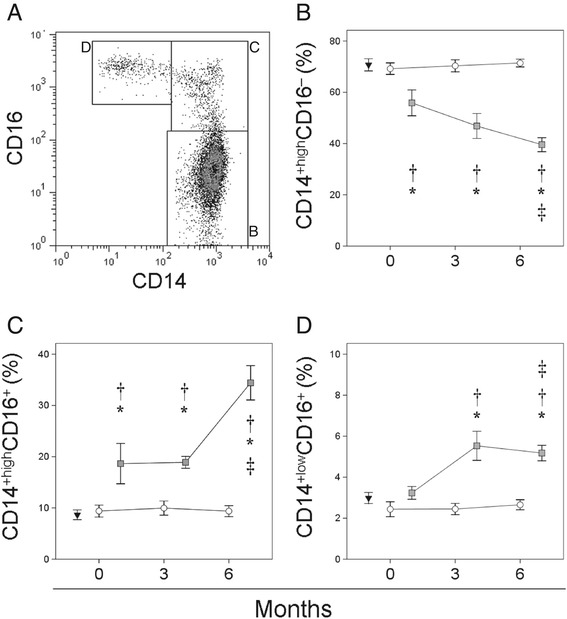
**Distribution of the CD14** ^**+high**^ **CD16** ^**−**^ **, CD14** ^**+high**^ **CD16** ^**+**^ **and CD14** ^**+low**^ **CD16** ^**+**^ **monocyte subsets in patients with RA at baseline and over MTX treatment.** Panel **A** shows flow cytometry analysis results for circulating monocytes from a representative responder patient. Percentages of circulating CD14^+high^CD16^−^ (panel **B**), CD14^+high^CD16^+^ (panel **C**) and CD14^+low^CD16^+^ monocytes (panel **D**) in non-responders (■) and responders (○) at baseline and after 3 and 6 months of MTX treatment, and in healthy controls (▼), are shown as means ± SEM. *Significant difference between patients with RA and healthy controls. ^†^Significant difference between non-responders and responders. ^‡^Significant difference between baseline and 6 month values.

Figure 2 shows the distribution of the CD14^+high^CD16^−^, CD14^+high^CD16^+^ and CD14^+low^CD16^+^ monocyte subsets in the patients and healthy controls. The non-responders showed a significantly smaller percentage of CD14^+high^CD16^−^ monocytes at baseline that further decreased over the 6 months of treatment with respect to responders and healthy donors. In contrast, the percentage of CD14^+high^CD16^+^ monocytes was significantly higher in non-responders at baseline and significantly increased during treatment with respect to responders. A different behavior was shown by the CD14^+low^CD16^+^ monocytes, the population of which expanded after 3 months of treatment in non-responders. No significant differences were seen in the distribution of the different monocytes subsets between responders and healthy donors.

### The number of circulating monocytes, and the numbers of CD14^+high^CD16^−^ and CD14^+high^CD16^+^ subset cells, predict the clinical response to MTX treatment in treatment-naive patients with RA

**Figure 3:**
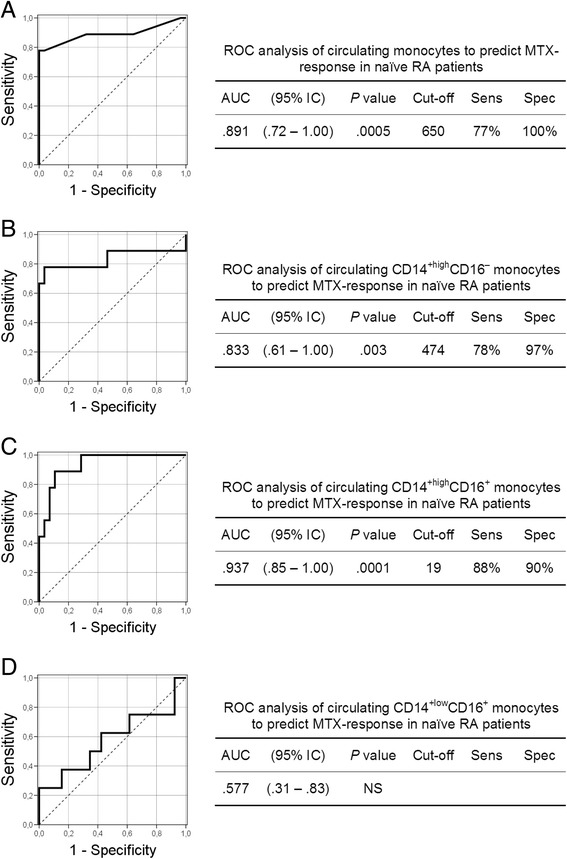
**Receiver-operating characteristic (** ***ROC*** **) analysis of the absolute numbers of circulating monocytes and of their subset cells.** Receiver-operating characteristic (*ROC*) analysis of the absolute numbers of circulating monocytes, and of the numbers of CD14^+high^CD16^−^, CD14^+high^CD16^+^ and CD14^+low^CD16^+^ subset cells (**A, B, C** and **D**, respectively). The predictive value of the absolute numbers of monocytes was determined by calculating the area under the curve (AUC). The optimum cut-offs (cells/μl) for distinguishing between MTX responders and non-responders, plus their sensitivity (Sens), specificity (Spec) values, are shown next to the curves. These were used to verify the validation of the ROC curves and to establish the predictive power of the cut-offs.

Figure 3 shows the predictive value of the absolute number of circulating monocytes, and of the numbers of CD14^+high^CD16^−^, CD14^+high^CD16^+^ and CD14^+low^CD16^+^ subset cells, with respect to clinical response to MTX. At baseline, a cut-off value of 650 cells/μl for circulating monocytes showed 77% sensitivity and 100% specificity in terms of discriminating between eventual responders and non-responders. A cut-off value of 474 cells/μl for the CD14^+high^CD16^−^ subset, and of 19 cells/μl for the CD14^+high^CD16^+^ subset, respectively showed 78% and 88% sensitivity and 97% and 90% specificity in terms of discriminating between eventual responders and non-responders. No significant differences were observed in the CD14^+low^CD16^+^ monocyte subset.

### CX3CR1 expression is increased in monocytes of non-responders

**Figure 4:**
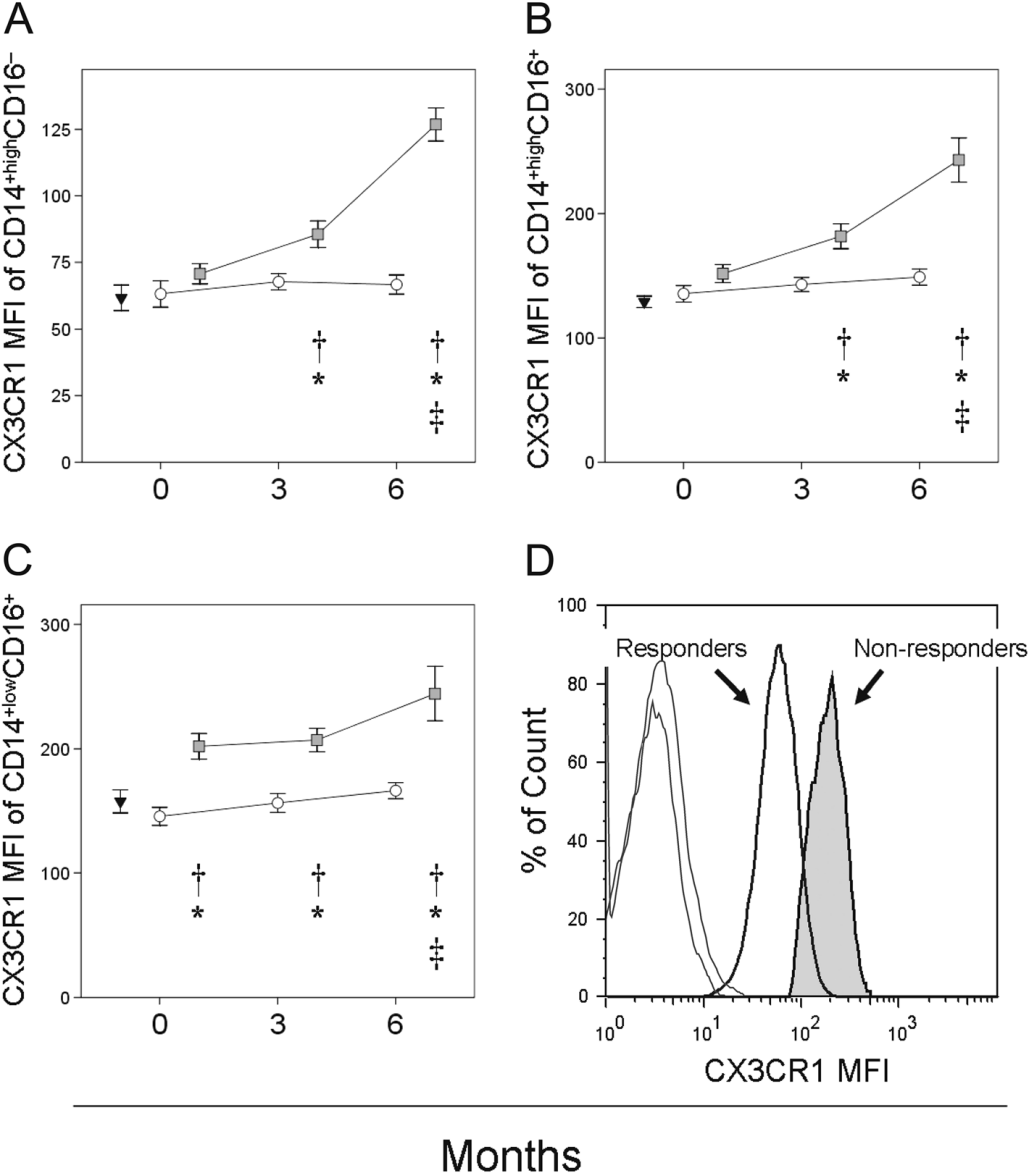
**Expression of CX3CR1 in the three circulating monocyte subsets examined in patients with RA at baseline and over MTX treatment.** Fluorescence intensities (MFI) of CX3CR1 in circulating CD14^+high^CD16^−^ (panel **A**), CD14^+high^CD16^+^ (panel **B**) and CD14^+low^CD16^+^ monocytes (panel **C**) of non-responders (■)and responders (○) at baseline and after 3 and 6 months of MTX treatment, and in healthy controls (▼), are shown as means ± SEM. Panel **D** is a histogram of CX3CR1 for circulating CD14^+high^CD16- monocytes from a representative responder and non-responder at 6 months of treatment and their respective isotype controls. *Significant difference between patients and healthy controls. ^†^Significant difference between non-responders and responders. ^‡^Significant difference between baseline and 6 month values.

The expression of CX3CR1 was determined in the three monocyte subsets in both patients and healthy controls (Figure 4). At baseline, the expression of CX3CR1 in CD14^+high^CD16^−^ and CD14^+high^CD16^+^ monocytes from MTX responder and non-responders was similar, and showed no significant differences with respect to healthy controls. A progressive and significant increase in CX3CR1 expression was observed in both monocyte subsets in non-responders after 3 and 6 months of treatment. In contrast, no significant differences in the CX3CR1 expression were seen between CD14^+high^CD16^−^ and CD14^+high^CD16^+^ monocytes from responders.

CD14^+low^CD16^+^ monocytes from non-responders showed increased CX3CR1 expression before starting treatment and during follow-up. In contrast, CX3CR1 expression in CD14^+low^CD16^+^ monocytes remained normal in responders throughout the study period.

## Discussion

This work shows that two different patterns of clinical response to MTX may be defined in treatment-naive patients with RA according to the features of the circulating monocyte subsets. The absolute number of circulating monocytes, and the numbers of CD14^+high^CD16^−^, CD14^+high^CD16^+^ and CD14^+low^CD16^+^ subset cells, provide good (high specificity and sensitivity) predictive biomarkers of the clinical response.

The treatment of RA has improved dramatically in recent decades. The introduction and routine use of MTX as a pivotal DMARD has provided enormous benefits to many patients [19]. However, not all patients profit from this treatment, although non-responders might enjoy marked clinical improvements with other drugs in the expanding armamentarium for RA treatment [20]. The identification of markers predictive of clinical response might, therefore, help limit the use of MTX on its own to those most likely to benefit from it, and allow likely non-responders to be given more appropriate treatment more quickly. This could be a major step forward in terms of treatment safety and in furthering the understanding of the factors that influence targeted therapy in RA.

Predictors of remission with DMARDs, such as male sex, shorter symptom duration or the absence of autoantibodies, have been suggested [21]. Recently, Ponchel *et al.* suggested that the baseline analysis of naive T-cells may help predict the response to MTX [22]. However, at present, no robust biomarker exits for rheumatologists to use in routine clinical practice that can predict responsiveness to this agent [14]. The present study therefore investigated the potential value of circulating monocytes for predicting the clinical response of treatment-naive patients with RA to MTX. This is justified by the critical role that monocytes and their cell subsets play in the induction of damage at inflamed joints and other tissue lesions [10]. The key inflammatory role of monocytes has been related to their ability to migrate to inflamed tissues, to provide effector functions such as cytokine and chemokine production, to undertake phagocytosis and oxidative radical generation, and to their ability to differentiate into different effector cells such as osteoclasts and dendritic cells [4,5]. It has also been shown that monocytes are important targets for MTX treatment in patients with RA [23,24]. It has been claimed that the different circulating CD14^+high^CD16^−^, CD14^+high^CD16^+^ and CD14^+low^CD16^+^ monocytes have different effector functions and vary in their capacity to differentiate into effector cells [6-8]. The present data show that the absolute number of circulating monocytes, and the numbers of CD14^+high^CD16^−^, CD14^+high^CD16^+^ and CD14^+low^CD16^+^ subset cells, are strongly predictive of the clinical response of naive patients with RA to MTX treatment. Interestingly, no differences were seen in the clinical characteristics of responders and non-responders in agreement with that reported in previous studies [25,26].

The present results also demonstrate the existence of two different patterns of numbers in the circulating monocyte compartment in treatment-naive patients with RA. In MTX responders, the number of cells in the monocyte subsets, plus the latters’ distribution, are similar to those found in healthy controls both before and during MTX treatment. In contrast, in MTX non-responders, a significant increase in the pre-treatment number of monocytes, and in the numbers of CD14^+high^CD16^−^ and CD14^+high^CD16^+^ subset cells, is seen. These numbers remain higher over the first 6 months of MTX treatment, while a significant increase in the number CD14^+low^CD16^+^ monocytes is observed after 3 months. These findings partially explain the controversial results reported for the numbers of circulating CD14^+high^CD16^+^ monocytes in patients with RA [12,27,28]. Interestingly, the different behavior of the circulating monocyte compartment shown by responders and non-responders cannot be ascribed to disease activity. Before starting MTX treatment, the activity of the disease was similar in both. It has been recently proposed that the CD14^+high^CD16^+^ monocyte subset participates in the expansion of T helper 17 lymphocytes found in RA patients [29]. Thus, the expansion of the CD14^+high^CD16^+^ monocyte subset might not only identify naive RA patients likely to respond poorly to MTX treatment, but also identify those in which a specific pathogenic mechanism is at work. Interestingly, the non-responders showed a significant redistribution in their circulating monocyte subsets over the treatment period, with a reduction in the percentage of CD14^+high^CD16^−^ monocytes. A reduction in this subset has been described in patients with RA after treatment with glucocorticoids [30,31]. It is interesting that the numbers of circulating CD14^+high^CD16^−^, CD14^+high^CD16^+^ and CD14^+low^CD16^+^ monocytes are also higher in patients with RA who do not respond to adalimumab plus MTX treatment [15]. Moreover, it has been found a correlation between the elimination of the expanded CD14^+bright^CD56^+^ monocyte subset in patients with RA and a good clinical response to TNF inhibiting agents [32]. These results support the idea that these cells might be important in the pathogenesis of RA and in the response to immunomodulator treatments.

The precise mechanism of action of MTX in patients with RA remains elusive [33]. It may act by reducing cell proliferation, by increasing the rate of leukocyte apoptosis, by increasing endogenous adenosine concentrations, or by altering cytokine production [24,34]. However, MTX is not a general anti-proliferative drug; indeed it induces apoptosis only in highly activated immune system cells [24,35]. The present data indicate that it causes no important modifications in the normal or increased number of circulating monocytes in responders and non-responders, respectively. Thus, its mechanism of action would not appear to involve a reduction in the production and survival of monocytes.

The present results show that MTX non-responder patients experienced variations in the monocytic subpopulations over the 6 months of treatment. The absence of a clinical response to MTX does not, however rule out the drug having biological effects on patient monocytes. Further, the progression of uncontrolled disease may be related to the alteration in monocyte subset cell numbers. Determining whether this is the case is impossible since is would be unethical to maintain patients with active RA without treatment. Moreover, the behavior of circulating monocytes in adalimumab non-responders is different to that observed in MTX non-responders [15].

It has been described that MTX can downregulate cytokine production and the expression of membrane receptors in monocytes in patients with RA [36]. The present results also show that the expression of the CX3CR1 migration receptors in monocytes is refractory to MTX treatment. Moreover, a large increase in CX3CR1 expression in the three different monocyte subsets was observed after 3 months of MTX treatment in the non-responders. The lack of effect of MTX treatment on monocyte CX3CR1 expression is further supported by the absence of modification of its constitutively increased expression in CD14^+low^CD16^+^ monocytes in non-responders. Future studies should aim to clarify whether the expansion of monocytes observed in MTX non-responders is associated with the abnormal function of these cells.

The observed predictive value of the absolute number of circulating monocytes, and of the numbers of CD14^+high^CD16^−^, CD14^+high^CD16^+^ and CD14^+low^CD16^+^ subset cells, in terms of clinical response to MTX treatment in treatment-naive patients with RA requires confirmation in large multicenter studies including patients of different race. However, the absolute number of monocytes, and the numbers of CD14^+high^CD16^−^, CD14^+high^CD16^+^ and CD14^+low^CD16^+^ subset cells, in peripheral blood would appear to provide practical biomarkers for predicting the response to MTX of treatment-naive patients with RA.

## Conclusions

In our work, we have investigated monocyte biomarkers as predictive factors for the response to methotrexate in rheumatoid arthritis. We have demonstrate that the absolute number of circulating monocytes and their CD14^+high^CD16^−^ and CD14^+high^CD16^+^ subsets have a predictive value for the clinical response to methotrexate treatment in untreated rheumatoid arthritis patients with high sensitivity and specificity. In addition, we also found a progressive redistribution of the monocyte subsets along 3 and 6 months methotrexate treatment in non-responder patients. These results have potential clinical relevance in the therapeutic management of rheumatoid arthritis and also contribute to the knowledge of the heterogeneity in its pathogenic mechanisms.

## Additional file

Additional file 1: Figure S1. Flow cytometry analysis of monocyte subsets. In the FSC-SSC dot plot, a biparametric gate was drawn around the monocyte population. This gated population is showed in a CD14-CD16 dot-plot to define the different monocyte subsets: “classic” monocytes (CD14^+high^CD16^-^); intermediate monocytes (CD14^+high^CD16^+^) and non-classical monocytes (CD14^+low^CD16^+^).

## Abbreviations

MTX: Methotrexate
RA: Rheumatoid arthritis
TNF: Tumor necrosis factor
EULAR: European League against rheumatism
DAS28: Disease activity score 28
DMARDS: Disease-modifying antirheumatic drugs
HAQ: Health assessment questionnaire
PBMC: Peripheral blood mononuclear cells
MoAbs: Monoclonal antibodies
SD: Standard deviation
SEM: Standard error of the mean
ROC: Receiver operating characteristic
AUC: Areas under the curves
